# Successful management of warfarin-exacerbated diffuse alveolar hemorrhage using an extracorporeal membrane oxygenation

**DOI:** 10.1186/2049-6958-8-16

**Published:** 2013-02-27

**Authors:** Jong Hoo Lee, Su Wan Kim

**Affiliations:** 1 Department of Pulmonary and Critical Care Medicine, Jeju National University Hospital, Jeju National University School of Medicine, Jeju, South Korea; 2 Department of Thoracic and Cardiovascular Surgery, Jeju National University Hospital, Jeju National University School of Medicine, Aran 13 gil 15, Jeju-si, 690-767, Jeju Special Self-Governing Province, Korea

**Keywords:** Alveolar hemorrhage, Anticoagulation, Extracorporeal membrane oxygenation, Hypoxia, Pulmonary thromboembolism

## Abstract

Although diffuse alveolar hemorrhage complicating warfarin therapy is rare, it generally has a worsening clinical course and can be a life threatening condition. A 56-year-old male who had undergone a pulmonary lobectomy for lung cancer 2 years before had received warfarin for about 5 months due to pulmonary vein thrombosis. The patient presented with severe dyspnea and had prolonged anticoagulation values. Chest X-ray and computed tomography revealed diffuse pulmonary consolidations, and bronchoalveolar lavage demonstrated diffuse alveolar hemorrhage. The reversal of anticoagulation was initiated, and extracorporeal membrane oxygenation was performed for refractory respiratory failure that did not improve despite maximal mechanical ventilatory support. The diffuse alveolar infiltrations resolved after 5 days, and we successfully weaned off both extracorporeal membrane oxygenation and mechanical ventilation. Herein we report the detailed course of a case that was successfully treated with extracorporeal membrane oxygenation as a bridge-to-recovery for warfarin- exacerbated diffuse alveolar hemorrhage.

## Background

Diffuse alveolar hemorrhage (DAH) refers to a clinical syndrome, resulting from extensive bleeding in the acinar portion of the lung [1]. It often presents with clinical features of hemoptysis, anemia, diffuse radiographic alveolar consolidation, and acute respiratory failure [1]. It is caused by diseases that damage the alveolar capillary barrier or by disorders of coagulation. These underlying diseases include: lung infections, pulmonary embolism, Wegener’s granulomatosis, Goodpasture’s syndrome, systemic lupus erythematosus and Behçet’s syndrome [2]. Only a few reports have mentioned the manifestation of alveolar hemorrhage in patients receiving anticoagulants, such as warfarin and heparin [3]. Furthermore, DAH complicating warfarin therapy is associated with a high mortality rate [4].

Extracorporeal membrane oxygenation (ECMO) can be considered in patients with life-threatening acute respiratory failure, such as severe hypoxemia, uncompensated hypercapnia and the occurrence of excessively high end-inspiratory plateau pressures, despite adequate management with a mechanical ventilator [5]. However, to our knowledge, ECMO applied to patients with fatal DAH has not been reported in the literature.

We report a case of successful application of ECMO as a bridge-to-recovery therapy in a patient with fatal warfarin-exacerbated diffuse alveolar hemorrhage.

## Case presentation

A 56-year-old male was admitted to our hospital with aggravated dyspnea over the previous 24 hours. He had a past medical history of right upper lobectomy for lung adenocarcinoma two years before. The pathologic stage was T3N1Mo (TNM stage IIIA), and adjuvant chemotherapy with vinca alkaloid (vinorelbine) had been followed postoperatively. However, newly-developed brain metastasis and metastatic nodules in the superior segment of the right lower lobe, had been diagnosed six months after the lung resection. He had undergone a whole brain radiation therapy and concurrent palliative chemotherapy with variable sequential regimens (pemetrexed, erlotinib, gemcitabine/cisplatin, and docetaxel) till six months before the re-admission. During the subsequent follow up the patient had shown a stable clinical course.

The patient was incidentally detected with right superior pulmonary vein thrombosis on follow up chest computed tomography, five months before the re-admission, and complained intermittent right arm swelling at this time. For the pulmonary vein thrombosis, he had been taking 2.0 mg to 2.5 mg of warfarin for five months. Prothrombin times (International Normalized Ratio, INR) had been usually controlled within 1.8 to 3.3 (INR target range: around 2.0), and he had not been prescribed other drugs that affect vitamin K metabolism during the previous six months. At the initial physical examination, the patient was looking very acutely-ill and markedly dyspneic. He did not present other respiratory symptoms like cough and hemoptysis. His vital signs were as follows: blood pressure 130/80 mmHg, pulse rate 115 beats/min, respiratory rate 40/min and body temperature 36.1°C. Auscultation revealed diffuse inspiratory crackles over both lungs. Results of laboratory investigations are shown in Table 1. There was no evidence of renal or hepatic dysfunction or autoimmune diseases. Any infection, heart failure, and platelet dysfunction were not suspect from the laboratory findings.

**Table 1:** Results of the initial laboratory investigations

**Variables**	**Results**	**Variables**	**Results**
**Complete blood count test**		**Chemistry profile**	
White blood cell	11,800/ul	Creatinine	0.9 mg/dL
Hemoglobin	11.4 g/dL	Potassium	4.0 mmol/L
Platelet count	226,000/ul	Creatine phosphokinase	99 IU/L
		Alanine transaminase	28 IU/L
**Arterial blood gas analysis**		**Cardiac specific enzymes**	
pH	7.36		
PaO_2_	51.5 mmHg	Troponin-T	0.017 ng/mL
PaCO_2_	34.0 mmHg	CK-MB isoform	4.01 ng/mL
SaO_2_	84.2 %	N-terminal pro-BNP	1,870 pg/mL
**Coagulation panel**		**Autoimmune antibody**	
aPTT	56 sec	Proteinase 3-ANCA	Negative
PT (INR)	86.3 sec (6.93)	Anti-nuclear antibody	Negative
**C-reactive protein**	9.47 mg/dL	Myeloperoxidase anti-neutrophil Cytoplasmic autoantibody	Negative
**Procalcitonin**	0.26 mg/dL		

ANCA, anti-neutrophil cytoplasmic antibody; aPTT, activated partial thromboplastin time; BNP, B-type natriuretic peptide; CK, creatine phosphokinase.

**Figure 1:**
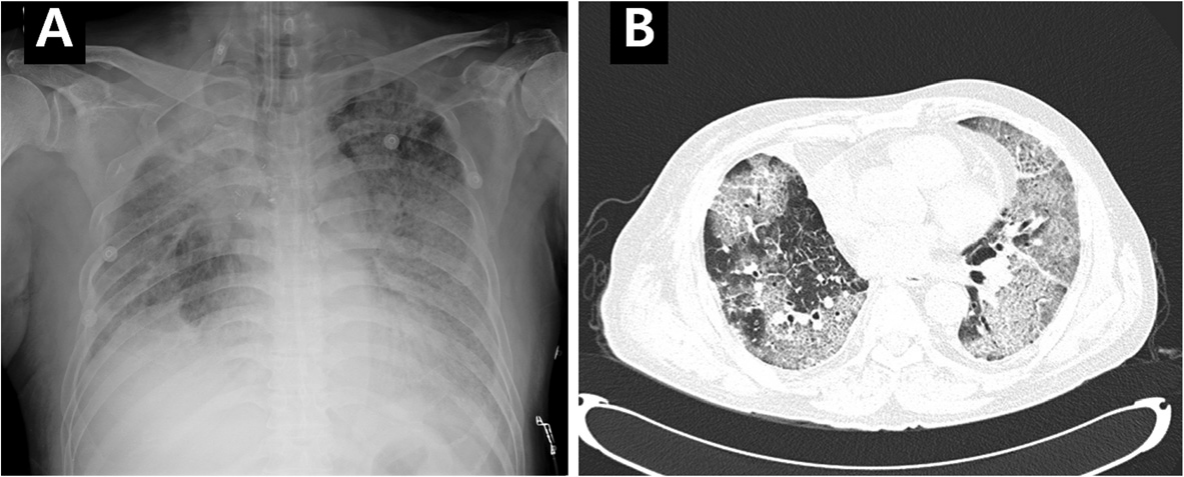
**Images at the time of admission.** Chest X-ray (**A**) and computed tomography (**B**) reveal bilateral pulmonary infiltrations and extensive ground-glass attenuations with crazy-paving appearance on whole lung fields.

A chest X-ray revealed increased bilateral pulmonary infiltrations, and chest computed tomography showed newly developed extensive ground-glass attenuations with crazy-paving appearance on whole lung fields (Figure 1). Ceftriaxone (2.0 g, once daily) and levofloxacin (750 mg, once daily) were empirically administered to prevent pneumonia. Warfarin was discontinued, and 10 mg vitamin K and 10 fresh frozen plasmas were administered intravenously. The deranged INR and activated partial thromboplastin time (aPTT) were corrected to 1.43 and 33 seconds respectively on the following day.

**Figure 2:**
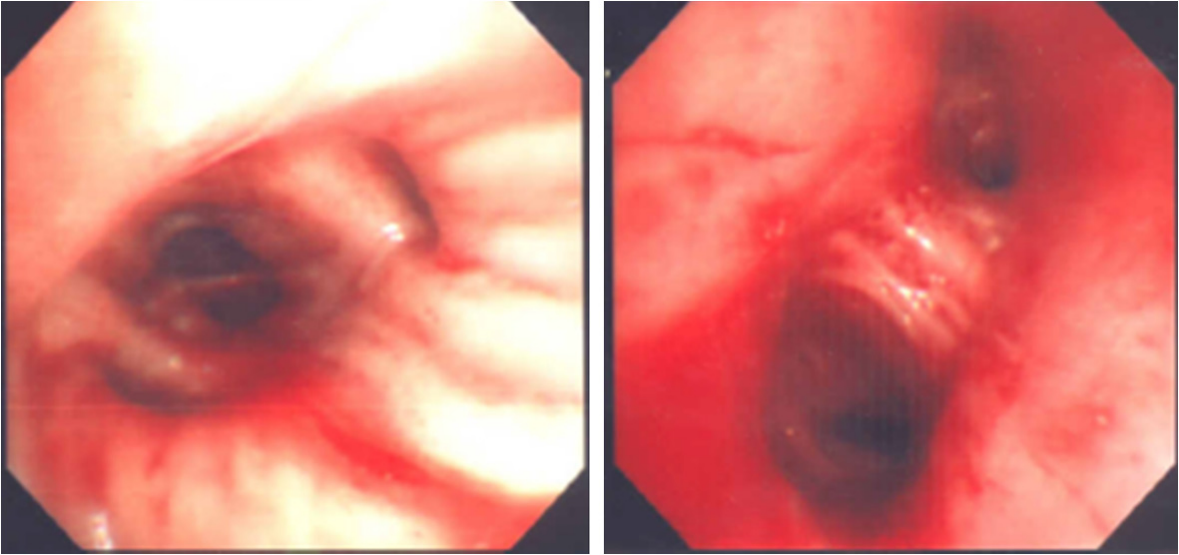
**Bronchoscopic findings.** Bronchoscopy shows fresh blood on whole bronchial system.

Since the patient’s oxygenation was not improved, despite maximum oxygen therapy through facial mask, endotracheal intubation and mechanical ventilation had to be started. Bloody secretions through the endotracheal tube were observed, and a flexible bronchoscopy was performed. Fresh blood on the whole bronchial system was observed, without any other endobronchial lesions (Figure 2). The bronchoscope was wedged into sub-segmental bronchi, and we confirmed the diagnosis of DAH by finding bronchoalveolar lavage (BAL) to become progressively more hemorrhagic. A cytopathological analysis with iron stains was not performed in the bronchoalveolar lavage (BAL) fluid. All cultures of sputum, BAL fluid and blood did not reveal any causative pathogens. The patient showed pure DAH confined to the lungs, and there was no evidence of disseminated intravascular coagulation or hemorrhagic events occurring elsewhere in the body.

In spite of maximal ventilatory supports (pressure control mode, a pressure support of 20 mmHg, at a rate of 20 breath/min, PEEP of 14 cmH_2_O and a FiO_2_ of 1.0), the patient remained severely hypoxemic (pH 7.36, PaCO_2_ 42.3 mmHg, PaO_2_ 48.4 mmHg and SaO_2_ 79.3%). Since the patient had very small safety margin in the remaining lung parenchyma, we decided to perform ECMO, instead of prone position, nitric oxide inhalation, and recruit maneuver.

**Figure 3:**
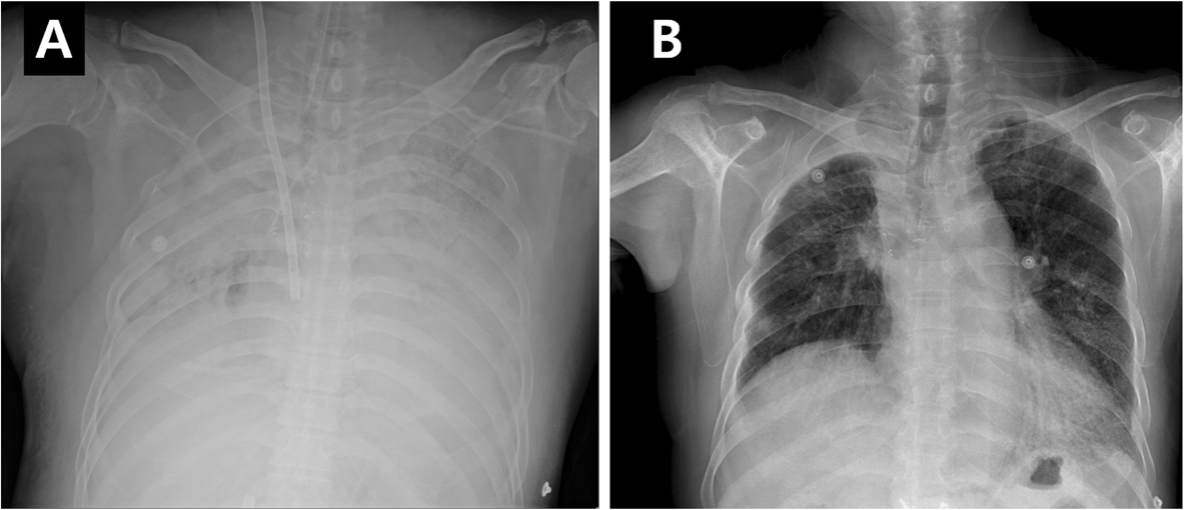
**Conduction of extracorporeal membrane oxygenation (ECMO).** Chest radiography shows diffuse pulmonary consolidations and a return cannula into the right internal jugular vein for ECMO (**A**), and improved bilateral pulmonary infiltrations after removal of ECMO (**B**).

A veno-venous type ECMO (EBS, Capiox® Emergency Bypass System, Terumo Inc., Tokyo, Japan) was applied to the patient on the day of hospitalization, with a 21-Fr drainage cannula from the left femoral vein and a 17-Fr return cannula into the right internal jugular vein (Figure 3A). ECMO was instituted without heparinization due to risk of alveolar hemorrhage. During ECMO, INR and aPTT of the patient were maintained within 1.25 to 1.43 and 21 to 33 seconds, respectively. Mechanical ventilatory care was minimally maintained (pressure control mode, a pressure support of 10 mmHg, at a rate of 10 breaths/min, a positive end-expiratory pressure of 10 cmH_2_O, and a FiO_2_ of 0.3) for lung protective strategy. Arterial blood gas analysis showed improved oxygenation and undisturbed gas exchange (Table 2), one hour after the application of ECMO. On day 5 after ECMO application, chest radiography showed diffuse pulmonary infiltrations were resolving (Figure 3B), and the oxygenation was improved, thus ECMO was removed. The duration of ECMO was 126 hours, and there was no complication associated with the procedure. An exchange of the oxygenator membrane was not needed, even though we did not use heparin. We performed extubation without tracheostomy 3 days after ECMO removal. the patient was discharged 42 days after the extubation and remained free from respiratory symptoms. We did not prescribe warfarin due to the concern of recurrent fatal DAH and the absence of symptoms of pulmonary or peripheral venous thrombosis. The patient has been controlled recently and is doing well.

**Table 2:** Changes in respiratory, ECMO, and coagulation variables

**Variables**	**Before ECMO**	**1 hour after ECMO**	**12 hours after ECMO**	**5 days after ECMO**	**1 day after ECMO removal**
pH	7.36	7.49	7.41	7.46	7.45
PaCO_2_ (mmHg)	42.3	27.8	38.6	44.6	40.4
PaO_2_ (mmHg)	48.4	59.0	75.9	153.0	77.2
SaO_2_ (%)	79.3	91.4	95.1	99.1	95.1
FiO_2_ in ECMO (%)	-	100	100	50	-
Blood flow in ECMO (L/min)	-	4.0	4.0	3.5	-
Tidal volume (ml)	240	200	260	380	480
Peak inspiratory pressure (cmH_2_O)	34	20	20	20	18
PEEP (cmH_2_O)	14	10	10	8	6
FiO_2_ in ventilator (%)	100	30	30	50	40
aPTT (sec)	56	-	33	27	21
PT (INR)	6.93	-	1.43	1.25	1.29

ECMO, Extracorporeal membrane oxygenation.

## Discussion

DAH is the result of the accumulation of intra-alveolar red blood cells originating from the alveolar capillaries [1]. Generally, DAH may develop in several pathologic conditions, including various vasculitis, autoimmune diseases, acute post-streptococcal glomerulonephritis, and potentially any pneumorenal syndrome. Uncommonly, drugs, such as inhaled cocaine, diphenyl-hydantoin, sirolimus, leflunomide, and everolimus, can also lead to DAH [6].

Warfarin (Coumadin) is a frequently prescribed oral anticoagulant inhibiting vitamin K utilization by the liver cells. Systemic bleeding events can occur in any organ or tissue, including the brain, and gastrointestinal or genitourinary tracts. However, the rate of alveolar hemorrhage in patients taking anticoagulant therapy is low and forms a minor part of bleeding complications [7-9]. DAH is a rare complication of warfarin therapy. DAH occurs rarely with high dose taken by children and young adults for suicide [10]. Since the first case of DAH caused by warfarin intoxication reported by Brown *et al.*[11] in 1965, few other reports have been described in the literature [12]. Warfarin-induced DAH is usually severe and can be lethal [4]. In the future, the incidence of anticoagulants-induced DAH may increase because of the growth of elderly population, who need anticoagulants due to conditions, such as atrial fibrillation, cardiovascular diseases, and valvular heart diseases. Furthermore, the new anticoagulants, such as abciximab and epifibatide, are more potent, and DAH induced by these drugs has recently been recently reported [13,14].

Our patient had abruptly increased INR, PT and aPTT. Although we investigated other concomitant drugs, trauma, or infections, we could not find the etiology of the suddenly prolonged anticoagulation. We did not examine the genetic polymorphisms of cytochrome P-450 enzyme 2C9 (CYP2C9) and vitamin K epoxide reductase complex 1 (VKORC1), which can affect the management of warfarin therapy. CYP2C9 is a key enzyme in the hepatic metabolism of warfarin. On the other hand, VKORC1 has a role in maintaining sufficient vitamin K levels when dietary vitamin K is limited, and the VKORC1 homozygous mutation affects the high response to warfarin [15].

We would highlight two points from this case. Firstly, it is very important to confirm an early diagnosis in clinically suspected patients with DAH, because it can be lethal due to anemia and respiratory failure. BAL is the gold standard for the diagnosis, and progressively hemorrhagic BAL finding in serial samples or positive iron studies of BAL will confirm the diagnosis [1]. However, a careful approach is needed because bronchoscopy itself can cause massive pulmonary bleeding. Secondly, as DAH complicating warfarin therapy is associated with a high mortality rate, prompt therapy should be initiated. A cornerstone of the treatment was reversal of the anticoagulation. It is life-saving to reverse anticoagulation by giving vitamin K and fresh frozen plasma early. The other principle of treatment is to maintain the patient’s oxygenation through aggressive treatment, via mechanical ventilation or ECMO when needed. We decided to start ECMO as a bridge-to-recovery or rescue therapy to maintain oxygenation, and ECMO was instrumental in saving patient’s life. To our knowledge, there are no previous reports of ECMO applied to patients with warfarin-induced DAH.

ECMO is an extracorporeal circuit that directly oxygenates the red blood cells and removes carbon dioxide from the blood. In patients with respiratory failure, while the results of the previous clinical trials did not show any survival benefit using ECMO or extracorporeal carbon dioxide removal [16,17], the recent CESAR study (Conventional Ventilation or ECMO for Severe Adult Respiratory Failure) using modern ECMO technology reported favorable outcomes [18]. Nowadays, ECMO is considered a bridge-to-recovery therapy in patients with profound gas-exchange abnormalities despite mechanical ventilation. As results of clinical studies and experiences were accumulating the indications for ECMO application have been increased.

During ECMO, there is continuous contact between the circulating blood and the foreign surface of the circuit [19]. The hemostatic balance is shifted to hypercoagulability with patients and extracorporeal circuits and components at risk for thrombosis. In order to regain the loss of hemostatic balance and prevent thrombosis, administration of antithrombotic drug, such as heparin, is necessary [20]. Unfortunately, the use of heparin can result in bleeding in the systemic circulation and despite its use, clotting in the extracorporeal circuit. The bleeding and thrombosis that occur regularly during the course of ECMO can ultimately result in significant clinical complications. Surgical site bleeding, the most common source of bleeding, is reported in 6-32% of ECMO patients with the highest incidence occurring in patients who have had recent cardiac surgery [21]. Intracranial hemorrhage, the most potentially devastating bleeding complication occurs in 3-6% of patients. Hypoxemia, acidosis, and cardiovascular instability prior to initiation of ECMO, and coagulopathy all increase the risk for intracranial hemorrhage [22]. Many unanswered questions exist concerning therapies to maintain the hemostatic balance during ECMO, including the optimal combination, duration and dosage of antithrombotic agents needed to limit bleeding and thrombotic complications.

Our practice is to use venovenous type ECMO for adult patients with respiratory failure. An echocardiogram prior to ECMO should be performed to confirm ventricular function is preserved. Heparin is infused to maintain an activated clotting time (ACT) of 140–160 seconds for venovenous ECMO and 180–210 seconds for venoarterial ECMO. We do not use any anticoagulants with our patient because heparin should promote the alveolar hemorrhage, and the thrombotic complications would be less serious venovenous ECMO than in venoarterial ECMO. Otherwise, venovenous ECMO can lead to significant alveolar hemorrhage or worsening of pre-existing alveolar hemorrhage. This could be related to ECMO flow directly into the right heart and pulmonary artery with shear forces disrupting the capillary bed, and observed by pulmonary arterial cannulation. If hypoxemia could not be improved and right heart failure aggravated despite venovenous ECMO, additional cannulation for venous drainage should be performed or conversion to venoarterial ECMO should be mandatory.

In practice, patients undergoing ECMO generally are subjected to early tracheostomy because of the need for prolonged management of underlying lung diseases. However, we did not perform tracheostomy, considering the high reversibility of DAH, and the patient had a favorable outcome. In patients with ECMO, tracheostomy should be selectively performed after considering the rapid reversibility of the underlying lung disease.

## Conclusions

Although warfarin-exacerbated DAH is rare, it can lead to potentially fatal respiratory failure. If a patient with warfarinization shows over-anticoagulation, prompt diagnosis and aggressive treatment for DAH should be performed considering a possible warfarin-exacerbated hemorrhage.

## Consent

Written informed consent was obtained from the patient for publication of this case report and any accompanying images. A copy of the written consent is available for review by the Editor-in-Chief of this journal.

## Competing interests

The authors declare that they have no competing interests.
